# Supplementary material for: Cognitive and emotional effects of bilateral prefrontal anodal tDCS and high-frequency tRNS in schizophrenia: a randomized sham-controlled study
Source: Schizophrenia (Heidelb). 2026 Jan 13;12(1):28. doi: 10.1038/s41537-025-00720-z (PMC12949120; doi:10.1038/s41537-025-00720-z)
Supplement: Supplementary file 1 — SUPPLEMENTAL MATERIAL [file 41537_2025_720_MOESM1_ESM.docx]

***Meassures***

1. *Spatial working memory (SWM)*

The SWM task is a measure of working memory. The computerized version of the task starts with several colored squares (boxes) displayed on the screen. The aim is to identify one yellow 'token' in each of the boxes by employing a process of elimination and then utilizing them to populate an empty column on the right side of the screen. The number of boxes can be incrementally raised based on the test's difficulty level, with a maximum of 12 boxes shown for participants to examine. The color and location of the boxes are varied from one trial to another. We were interested in the strategy scores and errors as outcome measures. The total error is the number of times a box is selected by mistake. The strategy score is an estimate of the use of this strategy obtained by counting the number of times the participant starts a new search with a different box. A high score represents poor use of strategy, and a low score corresponds to effective use.

1. *Stockings of Cambridge (SOC)*

The SOC, based on the Tower of London test ^1^, evaluates executive function—specifically spatial planning—and assesses frontal lobe functions, making this task sensitive to impairments in conditions like dementia, ADHD, and schizophrenia ^2^. In this task, participants aim to move colored balls to match the target pattern in the fewest moves possible. During the task, the participant views two displays with three colored balls arranged to resemble stacks held in stockings or socks on a beam. At the bottom of the screen, there is a row of numbered boxes. The test administrator first demonstrates how to match the pattern in the upper display by moving the balls in the lower display, completing one example that requires a single move. The task progresses from simple one-move problems to complex multi-move challenges, with a motor control phase to account for movement time, taking approximately 10 minutes to complete. Primary outcome measures included in this study were the mean number of problems solved (higher score better), and the mean number of moves to solve the problem for problem difficulty level (e.g., 2-move, 3-move, 4-move, etc.) (lower score better).

1. *Transcranial Direct Current Stimulation Side Effects Survey*

We used a standardized survey to evaluate the side effects of transcranial direct current and random noise stimulation (tDCS). Specifically, we employed the adverse effects questionnaire developed by Brunoni et al. (2011) ^3^, which provides a systematic framework for assessing tDCS-related side effects. This questionnaire includes 10 items—Itching, Tingling, Headache, Burning Sensation, Discomfort, Pain, Fatigue, Nausea, Skin Redness, and Difficulty Concentrating—each designed to capture the presence and severity of adverse effects experienced during or after tDCS sessions. Each item is rated on a Likert-type scale ranging from 0 to 5, where 0 indicates "Absent," 1 represents "Minimal," 2 is "Mild," 3 corresponds to "Moderate," 4 signifies "Marked," and 5 denotes "Severe." By standardizing the reporting of tDCS-related adverse effects, this questionnaire contributes to more consistent and comprehensive evaluations of tDCS safety in future research.

**References**

1. Langley C, Sahakian BJ, Robbins TW. Cambridge Neuropsychological Test Automated Battery (CANTAB). *The SAGE Handbook of Clinical Neuropsychology: Clinical Neuropsychological Assessment and Diagnosis* 2023: 435.

2. Owen AM, Downes JJ, Sahakian BJ, Polkey CE, Robbins TW. Planning and spatial working memory following frontal lobe lesions in man. *Neuropsychologia* 1990; **28**(10): 1021-34.

3. Brunoni AR, Amadera J, Berbel B, Volz MS, Rizzerio BG, Fregni F. A systematic review on reporting and assessment of adverse effects associated with transcranial direct current stimulation. *Int J Neuropsychopharmacol* 2011; **14**(8): 1133-45.
